# Supplementary material for: Structural insights into modulation and selectivity of transsynaptic neurexin–LRRTM interaction
Source: Nat Commun. 2018 Sep 27;9:3964. doi: 10.1038/s41467-018-06333-8 (PMC6160412; doi:10.1038/s41467-018-06333-8)
Supplement: Supplementary file 1 — Supplementary Information [file 41467_2018_6333_MOESM1_ESM.pdf]

Supplementary Information

Structural insights into modulation and selectivity of transsynaptic neurexin–LRRTM interaction

Yamagata *et al.*

a

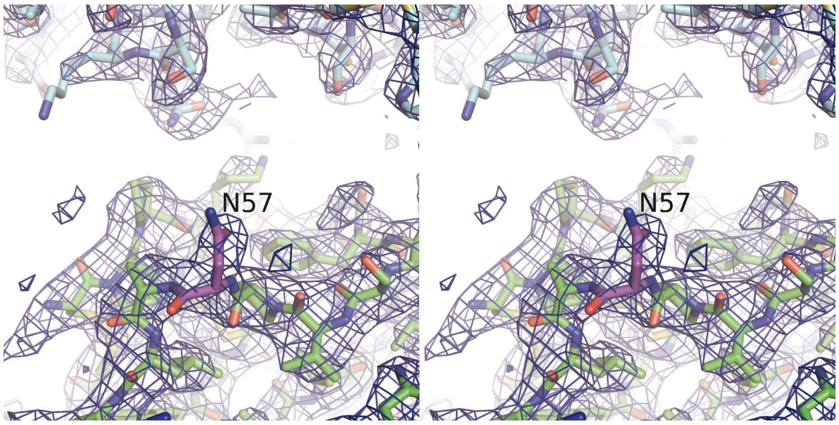

b

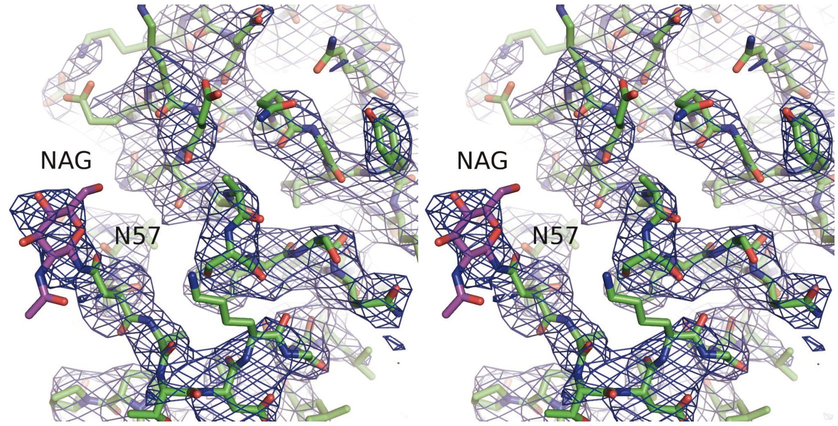

Supplementary Figure 1 Crystal contact of LRRTM2^{T59L} near Asn57.

(a) Stereoview of a crystal contact of apo-LRRTM^{T59L}. Two adjacent LRRTM^{T59L} molecules are shown as sticks. One is colored in cyan and the other is colored green, except that Asn57 is highlighted in magenta. LRRTM2^{T59L} lacks *N*-glycan at Asn57, due to the mutation of the consensus motif -Asn^{57}-X-Thr^{59}- to -Asn^{57}-X-Leu^{59}-. An electron density map ($2F_o$–F_c; contoured at 1.0 σ level) is overlaid.

(b) Stereoview of a region around *N*-glycan attached to Asn57 in the Nrxn1β-bound LRRTM2^{H355A} structure. LRRTM2^{H355A} is colored green, except that the observed *N*-acetylglucosamin (NAG) is highlighted in magenta. An electron density map ($2F_o$–F_c; contoured at 1.0 σ level) is overlaid.

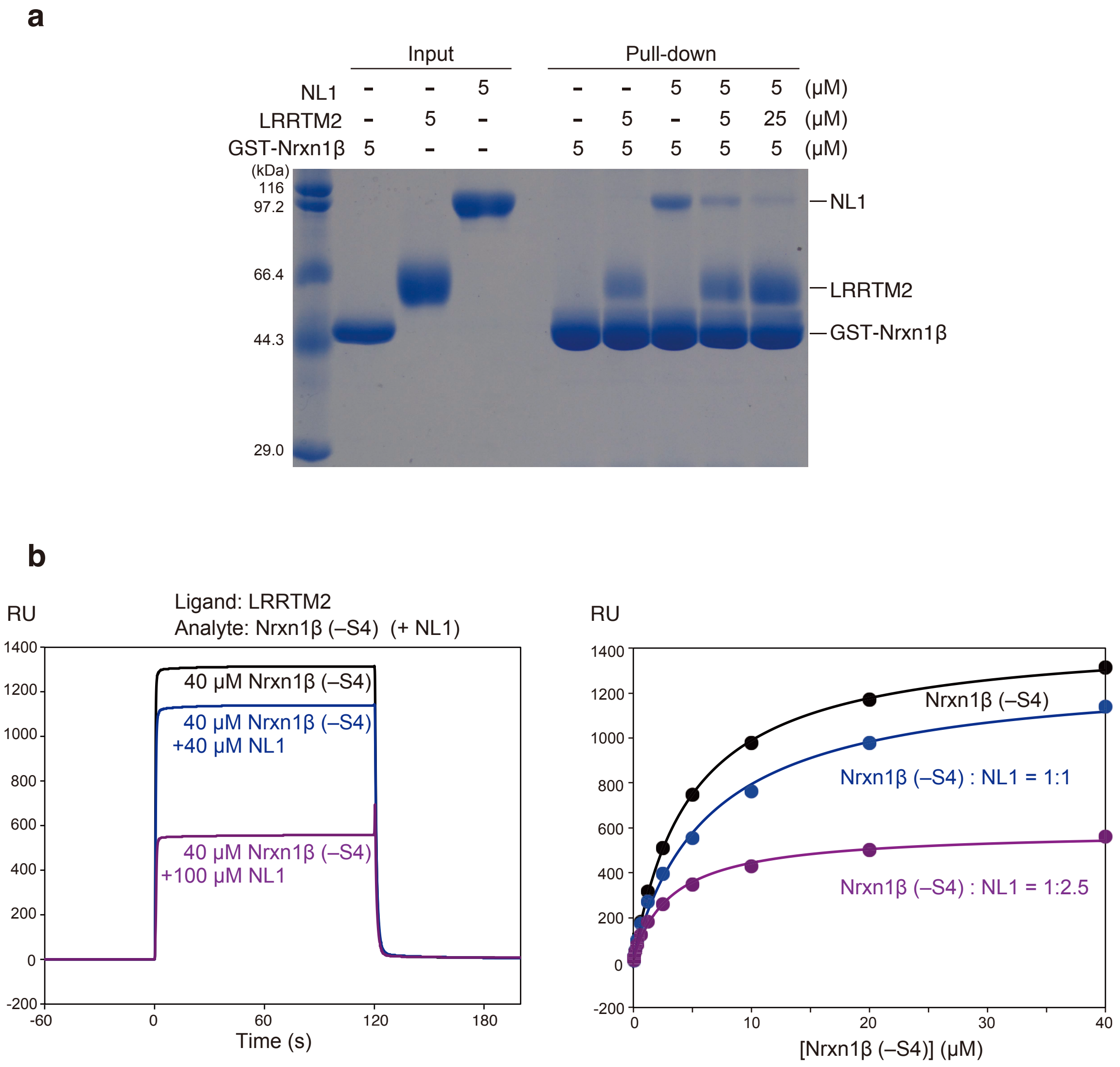

Supplementary Figure 2 Competition between LRRTM2 and NL1 for binding to Nrxn1β.

(a) Pull-down assay. GST-Nrxn1β, LRRTM2, and NL1 at the indicated concentrations were mixed and pulled down by glutathione beads. Input and bound proteins were analyzed by SDS-PAGE with Coomassie brilliant blue staining. **(b)** SPR analysis. SPR sensorgrams for injections of 40 μM Nrxn1β, 40 μM Nrxn1β + 40 μM NL1, and 40 μM Nrxn1β + 100 μM NL1 are overlaid (left). Equilibrium response units (RU) are plotted at different concentrations of Nrxn1β and Nrxn1β mixed with NL1 at molar ratios of 1:1 and 1:2.5 (right).

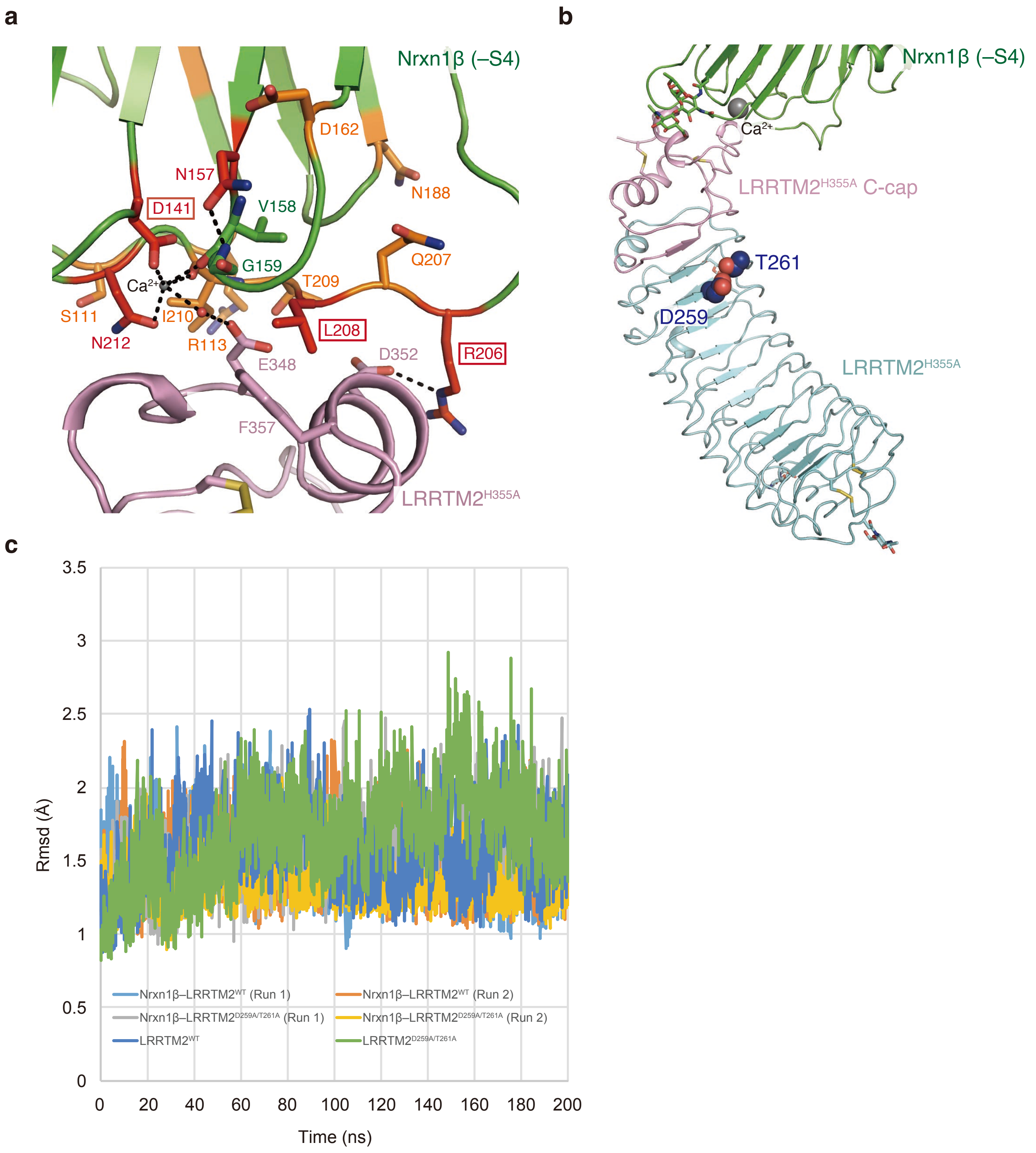

Supplementary Figure 3 Previously examined mutation sites in Nrxn1β and LRRTM2.

(a) Mapping of previously examined mutation sites in Nrxn1β on the Nrxn1β–LRRTM2^{H355A} structure. Mutation sites that critically or moderately affect the Nrxn1β–LRRTM2 binding are shown as red or orange sticks, respectively. The names of the residues that are also shown to be crtical by our SPR analysis are enclosed in red boxes. The coordinated Ca^{2+} and water molecule are shown as grey and red spheres, respectively. Dotted lines indicate hydrogen bonds.

(b) Mapping of previously examined mutation sites in LRRTM2 on the Nrxn1β –LRRTM2^{H355A} structure. Asp259 and Thr261 of LRRTM2 are shown as spheres.

(c) Rmsds from the initial structures during MD simulations (0–200 ns) of apo-LRRTM2^{WT}, apo-LRRTM2$^{D259A/T261A}$, and the Nrxn1β–LRRTM2^{WT} and Nrxn1β–LRRTM2$^{D259A/T261A}$ complexes.

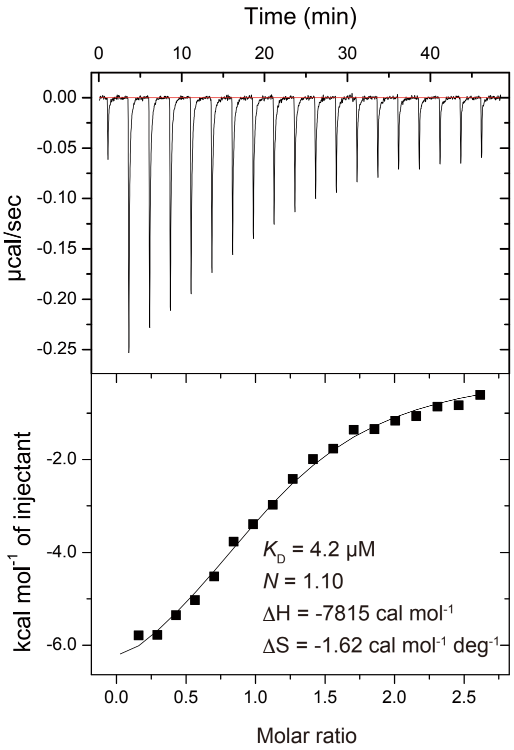

Supplementary Figure 4 ITC titration curve for binding between Nrxn1β and LRRTM2. The calculated thermodynamic parameters are also listed.

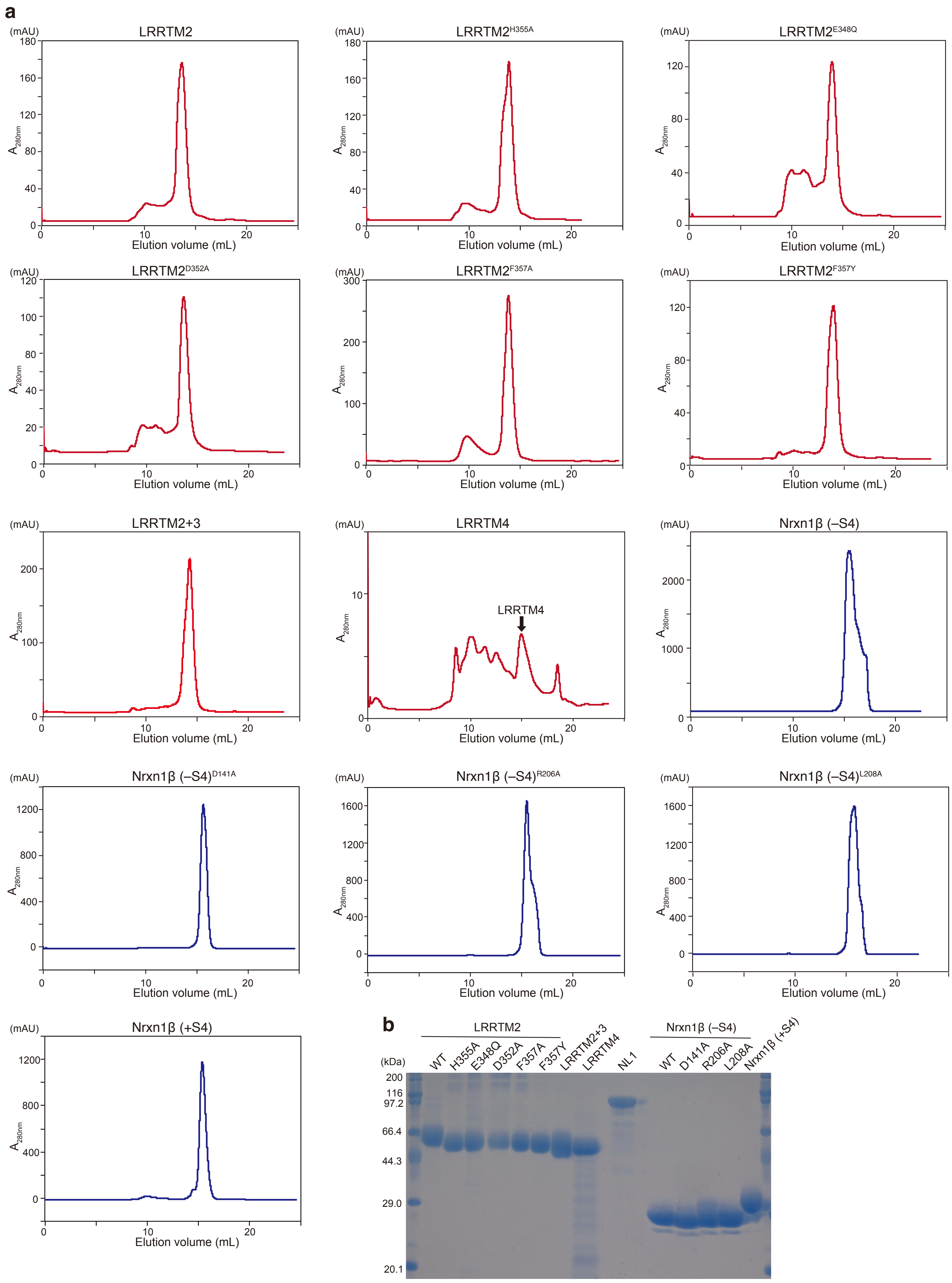

Supplementary Figure 5 SEC profile and SDS-PAGE of the samples used for SPR experiments

(a) SEC profiles of LRRTM2, LRRTM2+3, LRRTM4, and Nrxn1β samples purified by Ni-affinity chromatography.

(b) SDS-PAGE of the purified LRRTM2, LRRTM2+3, LRRTM4, NL1 and Nrxn1β samples.

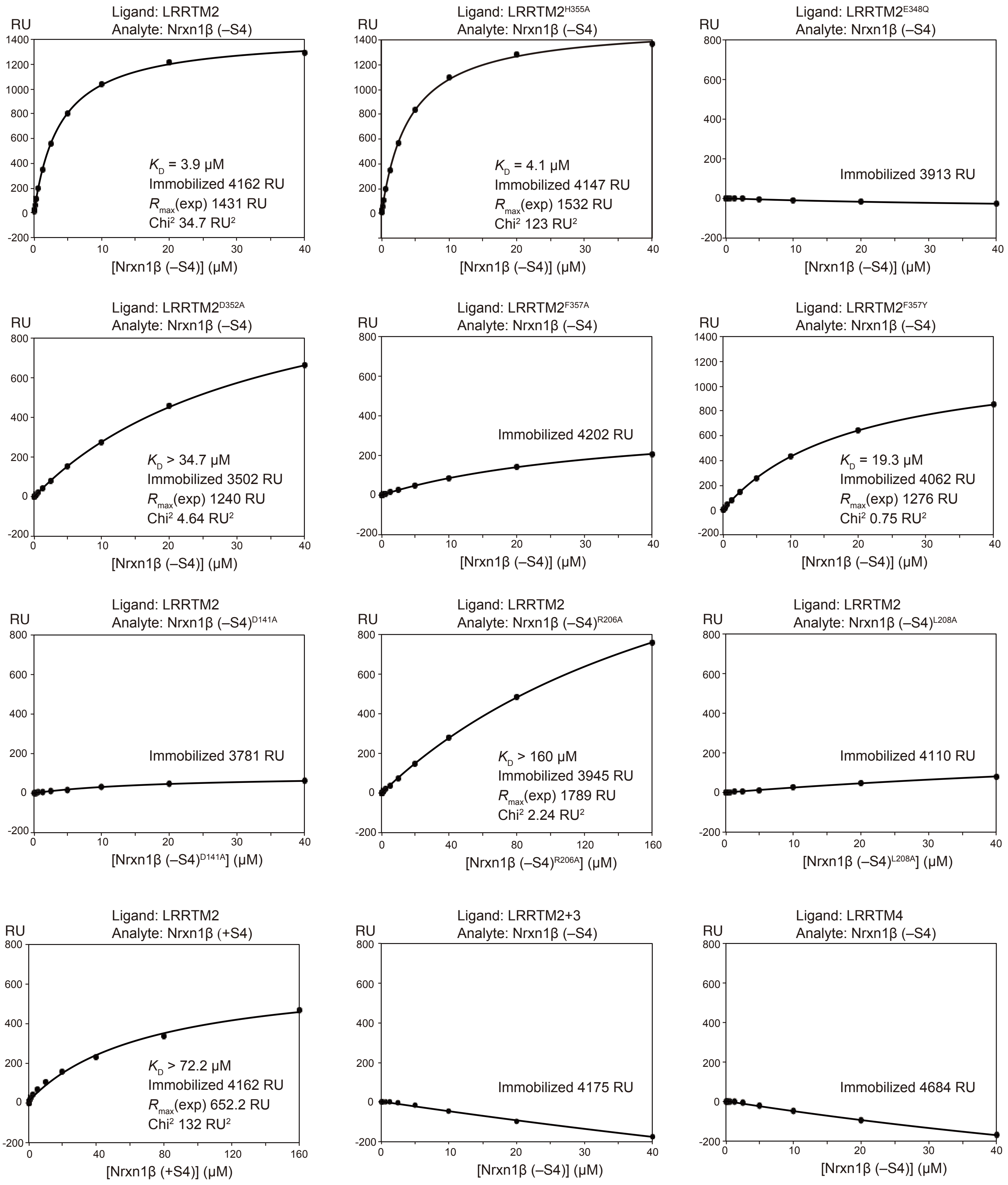

Supplementary Figure 6 Plot of equilibrium response units.

Equilibrium response units (RU) are plotted at different analyte concentrations. The amount of the immobilized ligand for each experiment is indicated. K_D, experimental R_{max} (R_{max}(exp)), and Chi^2 are also indicated for ligand–analyte pairs whose affinities can be calculated.

Supplementary Table 1 Rates of snapshots maintaining the Nrxn1β–Ca^{2+}–LRRTM2 interactions (< 3.5 Å) during MD simulations.

Pair		WT Run 1	WT Run 2	D259A/T261A Run 1	D259A/T261A Run 2
LRRTM2 D352@OD1,OD2	Nrxn1β R206@NE,NH1,NH2	0.756	0.993	0.884	0.714
LRRTM2 E348@OE1,OE2	Ca^{2+}	1.000	1.000	1.000	1.000
Nrxn1β D141@OD1	Ca^{2+}	1.000	0.997	1.000	1.000
Nrxn1β D141@OD2	Ca^{2+}	0.939	0.995	0.988	0.978
Nrxn1β I210@O	Ca^{2+}	1.000	1.000	1.000	1.000
Nrxn1β N212@OD1	Ca^{2+}	1.000	1.000	1.000	1.000

WT, Nrxn1β–$LRRTM2^{WT}$; D259A/T261A, Nrxn1β–$LRRTM2^{D259A/T261A}$
