# Supplementary material for: Structural insights into modulation and selectivity of transsynaptic neurexin–LRRTM interaction
Source: Nat Commun. 2018 Sep 27;9:3964. doi: 10.1038/s41467-018-06333-8 (PMC6160412; doi:10.1038/s41467-018-06333-8)
Supplement: Supplementary file 3 — Description of Additional Supplementary Files [file 41467_2018_6333_MOESM3_ESM.pdf]

Description of Additional Supplementary Files

File Name: Supplementary Data 1
Description: Primer sequences used in this study. The usage of each primer is also described.
